# The greatest challenges and solutions to improve children's health and well-being worldwide in the next decade and beyond: Using complex systems and implementation science approaches

**DOI:** 10.3389/fped.2023.1128642

**Published:** 2023-02-27

**Authors:** Zephanie Tyack

**Affiliations:** Australian Centre for Health Services Innovation and Centre for Healthcare Transformation, School of Public Health and Social Work, Queensland University of Technology, Brisbane, QLD, Australia

**Keywords:** implementation science, pediatrics, global challenges, complex systems, health, wellbeing

## Abstract

The health and well-being of children is paramount to health and well-being of society and is the foundation of health and well-being later in life. This paper presents the perspective that a complex systems approach that embeds implementation science is needed to address the rising challenges to child health and well-being in this decade (2020–2030) and beyond. Reflection on facilitators of the success of programs deemed promising to address child health and well-being in the past decade (2010–2020) is presented, to advance programs to address children’s health and well-being. A priority that needs to be addressed is developing, testing and using theories of child and family health and well-being (and related initiatives) that can be used to build on existing successes to make progress. Understanding context including further elucidating the drivers of child health and well-being at multiple levels of relevant systems (e.g., health, education, community) across the life course, and considering implications for caregivers also require greater consideration. Methods to address future challenges to child health and well-being include co-designing initiatives that support child health and well-being with children and families themselves rather than using predesigned initiatives, thoughtful outcome selection, and reporting the challenges of implementing future programs to promote learning. The approaches, priorities and methods presented can be used to design or refine interventions, models or care or community-based initiatives and provide new direction to fields of child health enquiry.

## Introduction

Agencies including UNICEF, WHO and UN Global Strategy for Women's, Children's and Adolescents’ Health recognise the health and well-being of children as being paramount to the health and well-being of the population worldwide (1–3). Childhood experiences and health are the foundation of health and well-being later in life (4) and in the coming decade children will need to respond to new and rising challenges. These challenges may include disasters, wars, food supply issues, epidemics and rapidly changing modes of communication which are likely to disproportionally affect women and children (5). An epidemic of mental ill-health, complex chronic disease, and lack of social connection and have been part of the health challenges for children and their caregivers over the last decade, which seems likely to continue and require urgent action (6–8). Despite wide-reaching impacts from children’s health on society, a lack of commitment to children’s health has been reported in some countries (9) and regression globally has occurred to an extent not seen in more than a generation (10). A complex systems with an embedded implementation approach is positioned as an important part of the solution to make progress in children’s health and well-being globally. This approach may assist to address key research gaps in understanding children’s health and well-being, and related multisector and multilevel initiatives that extend beyond the health sector including contextual factors or drivers influencing the health and well-being of children and the impact on caregivers and families. In addition, a better understanding of critical points of engagement that evaluation should target and translating the voice of children into new and existing initiatives may be achieved.

Children are referred to as those up to age 18 consistent with the Convention on the Rights of Children (11), although it is recognized that children younger than this age may make their own decisions regarding their health and treatment independent of guardians in some situations and countries. Whilst health and well-being are mentioned together for the sake of brevity as key concepts of focus, it is worth pointing out that the two are not synonymous and other concepts are interrelated. Debate and investigation of the conceptual and theoretical basis of each concept continue and multiple perspectives exist. An in-depth discussion of the different perspectives are beyond the scope of the paper. However, the understanding adopted for this paper and a few distinctions are worth noting (Table 1).

**Table 1:** Concepts, defining features and distinctions related to health and well-being.

Concepts related to health and well-being	Defining features and distinctions
Health	Health can be thought of as reflecting how a child feels about their relative (ideal versus actual) health states (12).
Well-being	Well-being is intentionally not defined in alignment with recently emerging qualitative studies that have identified the highly context-dependent, multi-faceted, changeable nature of child well-being; that requires lived out experience and meaning given by children that makes it difficult to quantify (13). A sense of what this may encompass can be obtained from qualitative studies of the meaning of well-being to children that have identified safety and security, social and personal relationships, adversity and hardship, vulnerability and agency, school success, and social spaces as important (13).
Objective versus subjective well-being	An emerging subjective approach to well-being differs to the predefined notions and domains targeting objective positive indicators of child well-being that arose as part of a major focus on quantitative survey research over the last two decades where positive indicators included the competencies, skills, behaviours, relationships and social connections, and the supportive environments that foster healthy development across the life of a child (14, 15).
Quality of life	Quality of life has been defined broadly as encompassing subjective well-being, health, health-related quality of life, and social indicators (12).
Health-related quality of life	Health-reported quality of life has been reported as a concept used mostly in patient populations receiving interventions (12).
Participation	Participation has been described as the day-to-day experience of health, well-being and a sense of belonging; and the means to achieving these experiences (16).

## Advances in personalising care and research gaps in the past decade

In the past decade advances have been made in advocacy for child, person-centred and family-centred models of health care involving children and families, that has included recognition of the need to personalise care and evaluation (17–19). Greater attention has been paid to mental ill-health in children and their parents and the social determinants of health (6) and advances have been made in understanding child development, prenatal care, nutrition, hygiene and family strengths (6, 14). We are on the cusp of a new wave of models of care and evaluation of initiatives based on input from children and families themselves, where recognition is given to the important role of coordinating and involving multiple disciplines and sectors (4).

Research gaps that require greater attention are the current disjointed consideration of physical, psychological, and social factors related to children’s health and well-being which are often examined separately; a lack of holistic care for children and families with complex conditions; and a lack of consideration of other contextual factors beyond the child that have a bearing on their health trajectory (for example, health of family members, intergenerational trauma, caregiver work-life balance, and the physical environment) (4, 6). The way health systems have evolved and operate means they are often inadequately equipped to provide holistic care that considers the child, family, treating health providers and community as a part of the whole system that works together to improve the outcomes of children (4, 17). Critical points of engagement with caregivers, engaging with the “community of care” for children in research (involving parents, grandparents, and other important people in children's lives), and systematically evaluating their participation and impacts on children, families and health systems are other areas that need to be addressed (18, 20).

The voice of children and families has been recognised as important in decision-making and communication with health professionals (16, 21) but more work is needed to translate this recognition into practice. For example, training health professionals regarding how to communicate, involving children in health decisions, and evaluating these initiatives (22). A recent review has indicated research initiatives in this area are increasing but work has predominantly been conducted in adult populations (23). Work is needed to identify progress in children’s participation in these initiatives which has been acknowledged as difficult due to a lack of monitoring data particularly at a country level (21).

The meaning of health and well-being (and related concepts like quality of life and participation) to children and their families is another area that requires theoretical elucidation which has been reported as under-developed (24, 25). The ramifications of this evidence gap are that if the conceptual meaning of terms including health and well-being (and related terms) are unknown or inadequately defined, measures used in research cannot be assumed to be valid or compared across studies (25).

## Understanding context

Awareness of the dynamic interaction of social-ecological, structural, cultural and environmental drivers of child, family and community health beyond the health system must continue to be considered and better understood to progress changes within relevant systems (for example, health, education) in this decade. Residential green space, air quality, screentime, sedentary living, consumerism, sleep habits, and neighbourhood noise have all been identified as impacting on children and families (6, 26). Socio-ecological factors of physical caregiving (providing supervision, meeting needs for food, parent work and lifestyle choices) and peer support (the presence of peers during challenges and feeling supported by peers) have been identified as helping to mitigate the negative effects of disaster thus bolstering resilience in children and youth (27). Understanding the important role of family routines (including working, sleeping, socialising and engaging with health services) which can impact on child and family well-being is needed as part of this (28).

Multiple perspectives of society, policy, and influences external to systems related to the health and well-being of children require consideration at the outset of developing and evaluating ongoing and new initiatives. These perspectives may include the child, family, education system, and community. The advantages of attending to multiple perspectives include influencing the level of awareness of key stakeholders, and intensifying scrutiny regarding access, availability and inequality of services (29). Prompting structural change and appropriate communication between policymakers and citizens can also be achieved (29). Equity can be influenced by attending to laws which may be changed to the disadvantage of children with disabilities and their caregivers, lack of political awareness of child inequalities, and poverty and economic inequality, for example, unemployment in single parent families deepening child poverty and hunger (29).

Reflecting on the facilitators of people, programs and countries who have made progress in child health in the last decade can provide direction to advance children’s health and well-being in the next decade. System-based child health initiatives reported as promising include those addressing population health to reduce inequity in whole developmental ecosystems such as All Children Thrive, Better Start Bradford and Generation Victoria (4) and a community health initiative involving collaboration between team members to support families’ accessing benefits, services, and legal protections, and identifying opportunities for system-wide improvement (30). A co-designed quality improvement initiative to improve antenatal care has also been implemented with women of refugee background and Australian-born women in four maternity hospitals that identified an increased proportion of refugee and Australian-born women attending the standard number of visits over time (22). Promise in these initiatives was indicated by success embedding elements such as a focus on the whole population, building equity into measures, and co-design with families rather than implementing predesigned programs (4, 30, 31). The in-depth understanding obtained regarding the positive and negative impacts of the programs, populations and outcomes that should be targeted in the future also indicated the promise of these initiatives (4, 30, 31).

## Approaches, priorities and methods

**Figure 1:**
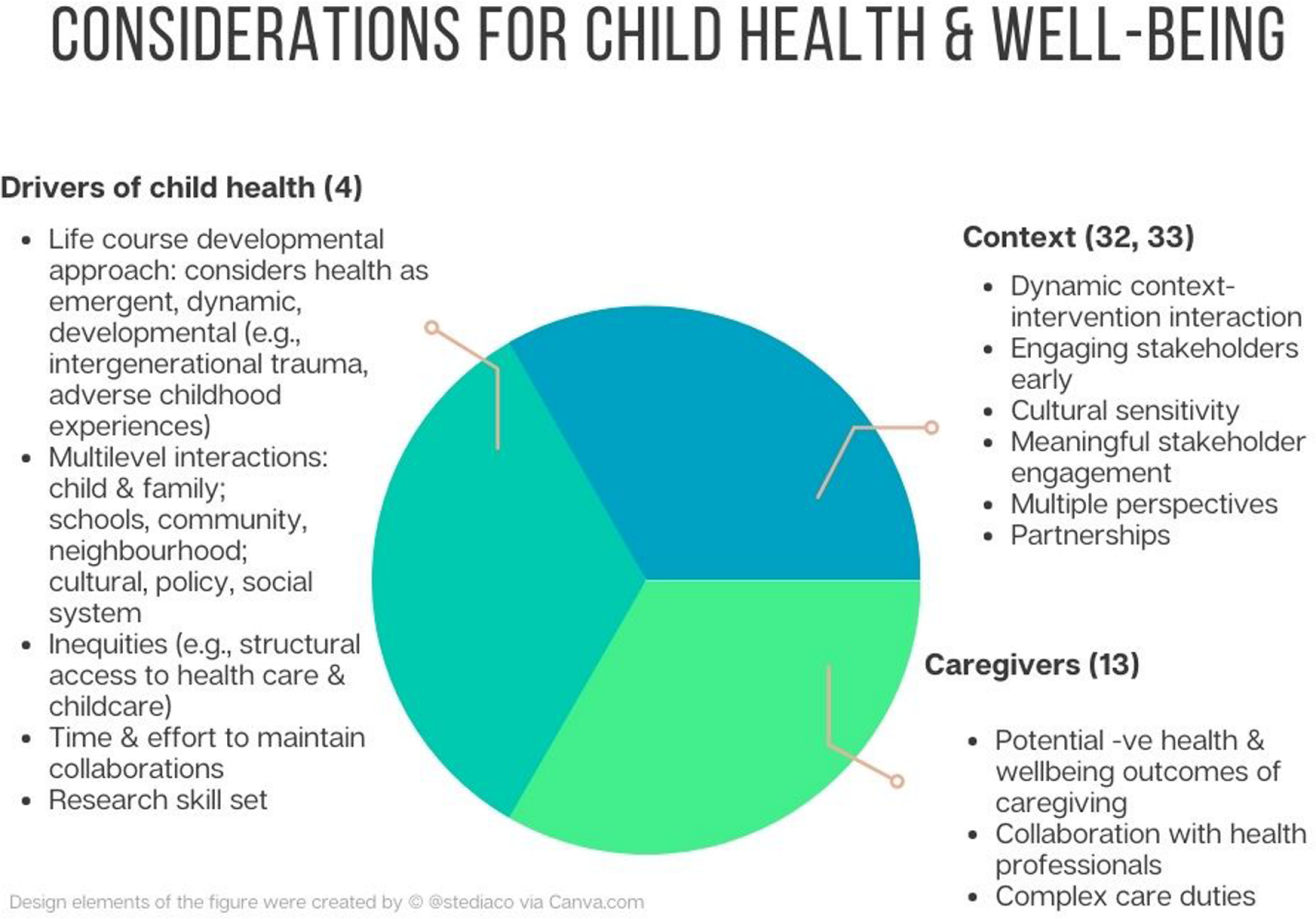
A complex systems approach.

A complex systems approach is needed to address challenges to the health and well-being of children and preventative health care of children in this decade. A complex systems approach positions health and inequality as outcomes of a complex interaction of elements within a connected whole system and uses a broad range of methods to design, implement and evaluate interventions, and models of care to improve health (32). Consideration needs to extend to the needs of communities (for example, housing, school, daycare availability) and families (for example, respectful communication between children, families and health professionals and collaboration to implement care plans) to improve and sustain health in children (18). A complex systems approach grounded in implementation science can assist in understanding the important role of context. This should include the social determinants of health in children (and impacts over a lifetime incorporating a life course health development approach) and contextual factors that influence developmental capability (for example, neighbourhood, family, school and health system factors) (4). The dynamic interaction between context, the implementation or de-implementation of interventions, and implementation strategies should also be considered (33). Figure 1 illustrates key considerations within a complex systems approach although these considerations should not be considered exhaustive.

A complex systems approach to the health of children and families should embed trauma-informed care. Reducing exposure to early childhood trauma and mitigating post-trauma effects has the potential to substantially reduce ill-health in adulthood, with reductions in the incidence of chronic obstructive pulmonary disease and depression estimated to be 27 percent and 44 percent respectively (34). Recommendations based on trauma-informed care principles have included: creating healthy communities; developing programs that apply primary, secondary and tertiary prevention to both reduce exposure to childhood adversity and mitigate the effects of trauma; and empowering patients and families by involving children and families in developing health and well-being policies and practices (34). Trauma-informed care should also target staff and health professionals in health systems with core principles that include recruiting and training a trauma-informed and compassionate workforce, creating a safe physical and emotional environment, and leadership commitment to trauma-informed care. Coordination of care across systems in the community including schools, juvenile justice, and mental health programs has also been recommended (34).

Determining priorities for implementing a complex system approach to child health in communities, health systems, and across countries is undeniably challenging due to the need to balance needs at each of these levels and across these areas. The need to balance early newborn care with prenatal and older child health has been discussed as a priority alongside the need to strengthen health systems and address social determinants of health and within-country disparity to move beyond survival of children worldwide (35). Influencing policy has also been highlighted as a priority (9). Balancing needs well has been discussed as having the potential to mitigate risks of disrupting routine perinatal and clinical care for children, worsening child health outcomes due to overburdened medical systems, and disrupting domestic economies and education systems (35).

Embedding implementation science in a complex systems approach to investigate child health and well-being can fast track the translation of evidence into practice building on existing programs and evidence (36). Implementation research is defined as “the scientific study of methods to promote the systematic uptake of proven clinical treatments, practices, organizational, and management interventions into routine practice, and hence improve health” (37). Robust implementation research has the potential to mitigate the epidemic of paediatric chronic disease (7), advance research into early child development (24), and fast track evidence of the effectiveness and implementation of family-centred models of care that have been developed (7). However, evidence to determine the core elements of implementation theories, models and frameworks for paediatric research and practice and how these frameworks can be adapted to embed equity is first needed to meet this objective (38, 39). Recognising and building on the strengths and limitations of existing implementation theories, models and frameworks is needed going forward, as well as drawing on more in-depth interpretive qualitative methods such as ethnography and grounded theory to develop new theory.

System-wide or multi-level theories, models or frameworks should be considered as part of complex systems and implementation research in child health evaluations and study designs (9). Examples of these include the socioecological model of factors influencing child and family health and well-being (6), and an ecological model of child development adapted from Bronfenbrenner (1979) (40). The latter places the individual in the centre, surrounded by four nested levels of influences including family and classroom, parents’ workplaces and individual’s ethnicity, culture or beliefs. The EPIS (Exploration, Preparation, Implementation, Sustainment) framework is another framework that considers multiple levels where implementation takes place (i.e., the individual, the organization, the community, the inner or outer setting) as well as factors that bridge those levels (for example, partnerships) (41) and has been applied in child, youth and family service settings (38). For interested readers a scoping review provides some guidance for selecting implementation frameworks in these child and family service settings, however the frameworks identified had limitations in that they were rarely theoretically grounded or fully developed, and none were developed or applied outside the US (38). Thus developing and refining theoretically grounded and fully developed theories are a priority going forward. While the use of existing models and frameworks may guide and structure understanding, an awareness of their potential limitations is needed in that they may miss important determinants and processes, as few were developed in paediatrics. While evidence is accumulating regarding conceptually and theoretically developed theories in paediatrics, the use of inductive qualitative methods alongside deductive framework based methods has merit. This would be a method of ensuring important determinants in implementation frameworks are not overlooked using deductive methods (42) and that determinants and processes not in existing models and frameworks are not missed using inductive methods.

Methods that should be applied are the convergent approaches of linking multiple sectors (for example, education, housing and social protection), multi-disciplines, community engagement, and co-produced knowledge (43) in order to advance the health and well-being of children in future decades. It is also crucial that population-based decision support systems and platforms are developed and used as part of this (44). These platforms can be used for prospective, multidimensional, deeply characterised cohorts, that include a multitude of data to support policy and planning, service delivery, personalised care, and public health (44). Methods that should continue to be applied to child health research as part of complex systems and implementation science approaches include mixed methods and participatory theory of change methods, recognising the challenges of applying these methods in the past (9, 31, 45). These challenges include difficulty being able to evaluate the changing nature of context and drivers of system change over time, evaluation within disjointed data systems, and developing a shared vision and values with those involved in partnerships to achieve system change (45). New outcome measures may also need to be developed and tested where suitable outcome measures do not exist [e.g., a family strengths measure (18)]. Challenges to the implementation of future programs and how these were managed should be reported as this can strengthen the quality of programs in the future with stronger scalability and sustainability (36).

The selection of outcomes in paediatric research and practice or prevention initiatives requires careful thought. When child clinical or health outcomes have been the focus of research aggregated group outcomes have often been the focus of the development of core outcome sets, rather than individualised child outcomes or meaningful outcomes that pertain to the individual goals and needs of the child or family (19, 46, 47). Meaningful outcome measures are needed that capture youth and family priorities and preferences, which then need to be prioritized or balanced across these stakeholder groups (25). For example, family perspectives may need to be prioritised when interventions and children of younger ages are involved (25). For initiatives where evidence is being translated or tested in real world settings, important outcomes related to implementation processes, and multiple levels involving families, communities and the health system that support child health, require careful consideration (46, 48). Modern connotations of child empowerment and autonomy need to be examined alongside diverse well-being outcomes (6). For example, the socialisation of children as consumers through media and parental agreement to dominant sociocultural ideologies of consumerism and materialism, can serve to reinforce poor choices by children themselves (e.g., large amounts of screentime, poor food choices and low physical activity) that puts their well-being at risk (6) thus outcomes reflecting child empowerment and autonomy may not be synonymous with child well-being. Importantly, older children themselves should be involved in reporting their outcomes where appropriate as there is growing evidence of children’s cognitive ability to meaningfully reflect on their lives, and to report on their subjective well-being (49).

## Discussion and conclusion

A complex systems approach that embeds implementation science is needed to advance child health and well-being in this decade and beyond. A priority using this approach is developing, refining and using substantive or mid-range theories of health and well-being and initiatives that apply to specific contexts as well as fully developed theory to support intervention development, implementation, and evaluation and be able to build upon existing program successes. Limitations of this priority may be the time, expertise and depth of analysis needed to develop these theories which may be in contrast to the rapid analysis methods commonly used in implementation science (50). Also without capturing relevant perspectives in theory development there is the risk that meaningful perspectives are not captured nor comprehensive understandings obtained, thus the reach and impact of the theory may be limited. For example, methodologies that take the perspective of a unit (for example, the nuclear family) may have limitations when multiple perspectives interact with, but are outside of, the unit of focus (for example, the school perspective). Consideration may first need to be given to methodologies that commit to depth of analysis but also capture the complexity of multiple perspectives using multiple perspective designs like interpretive phenomenological analysis (IPA) where the focus can be on the person, family or system (51). These multiple perspective designs can inform later theory development (52).

Understanding context including the drivers of child health and well-being at multiple levels of relevant systems and communities extending across the life course can be used to design interventions, models or care and community-based initiatives and provide new direction to fields of child health enquiry including public health, health services research, implementation science, sociology, genetics, and health economics. It will be important that diverse stakeholders including researchers, policy makers, managers in health services, health professionals, community members, teachers, caregivers, extended family members and children contribute to this important work and commit to raising the profile of children’s health for greatest impact.

## Data Availability

The original contributions presented in the study are included in the article/Supplementary Material, further inquiries can be directed to the corresponding author.
